# The Use of Cannabis as a Predictor of Early Onset of Bipolar Disorder and Suicide Attempts

**DOI:** 10.1155/2015/434127

**Published:** 2015-05-13

**Authors:** Rafaela Torres Portugal Leite, Sarah de Oliveira Nogueira, João Paulo Rodrigues do Nascimento, Laisa Soares de Lima, Taís Bastos da Nóbrega, Mariana da Silva Virgínio, Lucas Monte da Costa Moreno, Bruno Henrique Barbosa Sampaio, Fábio Gomes de Matos e Souza

**Affiliations:** ^1^Faculty of Medicine, University of Fortaleza, 221 Desembargador Floriano Benevides Street, 60811-905 Fortaleza, CE, Brazil; ^2^Department of Psychology, Federal University of Ceará, 2762 Universidade Avenida, 60020-181 Fortaleza, CE, Brazil; ^3^Faculty of Medicine, Christus University Center, 133 João Adolfo Gurgel Street, Fortaleza, CE, Brazil; ^4^Faculty of Medicine, Federal University of Ceará, 949 Alexandre Baraúna Street, 60430-160 Fortaleza, CE, Brazil; ^5^Department of Clinical Medicine, Federal University of Ceará, 1290 Capitão Francisco Pedro Street, 60430-140 Fortaleza, CE, Brazil

## Abstract

*Introduction*. Bipolar disorder (BD) implies risk of suicide. The age at onset (AAO) of BD carries prognostic significance. Substance abuse may precede the onset of BD and cannabis is the most common illicit drug used. The main goal of this study is to review the association of cannabis use as a risk factor for early onset of BD and for suicide attempts. *Materials and Methods*. PubMed database was searched for articles using key words “bipolar disorder,” “suicide attempts,” “cannabis,” “marijuana,” “early age at onset,” and “early onset.” *Results*. The following percentages in bipolar patients were found: suicide attempts 3.6–42%; suicide attempts and substance use 5–60%; suicide attempts and cannabis use 15–42%. An early AAO was associated with cannabis misuse. The mean age of the first manic episode in individuals with and without BD and cannabis use disorder (CUD) was 19.5 and 25.1 years, respectively. The first depressive episode was at 18.5 and 24.4 years, respectively. Individuals misusing cannabis showed increased risk of suicide. *Discussion*. Cannabis use is associated with increased risk of suicide attempts and with early AAO. However, the effect of cannabis at the AAO and suicide attempts is not clear.

## 1. Introduction

Bipolar disorder (BD) is associated with poor health outcomes and is responsible for the highest rate of suicide among all mental disorders [1]. Bipolar disorder is often complicated by cooccurring substance use disorders [1]. Cannabis is the most common illicit substance used among individuals with bipolar disorder [2] and up to 38% of the individuals with bipolar disorder misuse it [3]. Cannabis abuse has particularly been reported to be high among young bipolar patients [4], and chronic cannabis use is associated with higher severity of illness and greater noncompliance to treatment among individuals with bipolar disorder [5].

In bipolar disorder, there is evidence that, with many patients, substance abuse precedes the onset of BD, and it has been suggested that affective deregulation may increase the risk of bipolar disorder due to substance abuse [6]. The relationship between substance abuse and age at onset of bipolar disorder is not really well understood [7]. Among individuals with cooccurring CUD, age at onset of bipolar disorder was 6 years lower and the mean number of manic, hypomanic, and depressive episodes per year was greater compared to individuals without CUD [8].

Lev-Ran et al. [9] also reported that cannabis use may decrease the age at onset in both schizophrenia and BD. This is consistent with the view that cannabis use may unmask a preexisting genetic liability that is partly shared between patients with schizophrenia and bipolar disorder. The reduction was 9 years of the age at onset in bipolar group [9]. Lagerberg et al. [8] found a significant association indicating a dose-response relationship between cannabis use and age at onset, which remained statistically significant after controlling for possible confounders (gender, bipolar subtype, family history of severe mental illness, and alcohol or other substance use disorders). The mean difference in age at onset between the groups with and without cannabis use disorder in that study was 5 years.

It is important to review the definitions of age at onset, since these definitions are controversial in the psychiatric literature. Patients displaying bipolar disorder at a younger age at onset have higher prevalence rates of psychotic symptoms, substance abuse comorbidity, learning disabilities, and episodes of rapid cycling of mood [10, 11]. Moreover, there is an increased risk factor for suicide attempts [12].

Among common mental disorders, BD implies a particular risk of both nonfatal self-harm and completed suicide [13]. The risk of suicide in bipolar patients is 20–30 times higher than that of the general population [14]. The risk is greater among those who have been admitted to inpatient care due to bipolar disorder [15] and especially high in bipolar patients admitted to inpatient care after suicide attempts [16].

The main aims of this study are to review (1) the definition of the age at onset of BD, (2) the association of cannabis use as a risk factor for early onset of BD, and (3) the relationship of cannabis use and suicidal behavior in BD.

## 2. Materials and Methods

Between June and September 2014, PubMed database was searched for articles using combinations of the following key words: “bipolar disorder,” “suicide,” “suicide attempts,” “cannabis,” “marijuana,” “early age at onset,” and “early onset.” No language or publication time constraints were applied. PubMed database indexes articles published since 1948 up to the present date. Data specifically related to bipolar disorder were chosen. Duplicates and repetitive reviews were excluded. In the cases of similar studies performed by the same group, the study with bigger sample size was included in this review. The “related articles” function of the PubMed database, the reference list of selected articles, conference abstracts, and Google Scholar were also used to identify additional articles. This review follows the PRISMA guidelines.

The entry criteria of the articles included in this review should meet at least one of the following requirements: (1) the selected study should provide data that allowed evaluating the age at onset of bipolar disorder; (2) the paper should present the proportion of bipolar patients who used cannabis; (3) patients included in these studies should have had an early age at onset of BD or suicide attempts.

## 3. Results

Initially, for the keywords “early onset,” “bipolar disorder,” 1,017 articles were found; for “cannabis” and “bipolar disorder,” 143 articles; for “suicide attempts” and “bipolar disorder,” 1,069; for “suicide” and “bipolar disorder,” 2,289; for “marijuana” and “bipolar disorder,” 190; and for “early age at onset” and “bipolar disorder,” 626. 59 duplicates or repetitive reviews were excluded. Of all articles reviewed, 77 papers (all of them published) fulfilled the entry criteria and were included, as shown in the Table 1. They were published between 1994 and 2014, 40 of them (51%) in the last 5 years.

[Fig fig1]

### 3.1. Definition of Early Age at Onset of Bipolar Disorder

The age at which the first bipolar episode occurs is relevant because an early age is associated with poor prognosis [23]. If an early diagnosis of BD is not established, the treatment starts late, compromising its good outcome [34]. Subgroups defined as “early onset” and “very early onset” were associated with greater rates of comorbid anxiety disorders and substance abuse, violent behavior, rapid cycling, and shorter periods of euthymia compared to the “late onset” subgroup [23].

The DSM-IV suggests the age of 21, supported by genetic studies, as the maximum age to be included as the early age at onset of BD [35]. An epidemiological study with 61,392 community adults in 11 countries found that the mean age at onset of BD type I was 18.4 (±0.7) years, BD type II was 20.0 (±0.6) years, and subthreshold BD was 21.9 (±0.4) years [36]. Other studies described different definitions of early age at onset, being <21 years the most common age defined as “early onset,” as shown in Table 2.

Table 2 shows chronological studies with different definitions of early and late ages at onset. The following variables are described: year of publication; sample size; study design; and ages at very early, early, intermediate, and late onset. Sample sizes ranged from 169 to 1,856. Only one article establishes very early age at onset as <13 years. The definition of early age ranged 17.4–21, while late age ranged 18–40.

In a study comparing a sample of early (*n* = 58) and late onset (*n* = 39) bipolar patients, the early onset group had a more severe form of the disorder with more psychotic features, mixed episodes, comorbid panic disorder, and poorer response to lithium. The early onset group was defined as younger than 18 and late onset as older than 40 [10].

Another paper comprised 52 (50 BD type I, 2 BD type II) patients who had an early onset and 38 (30 BD type I, 8 BD type II) a late onset. It was observed that the early onset group is characterized by a higher frequency of psychotic symptoms as compared to patients with late onset [17].

A sample of 320 individuals diagnosed with BD I or II was stratified into subjects with early age (≤18 years) and late (>18 years) age at onset of BD. A significant earlier age at onset in subjects with anxiety disorders and rapid cycling course was found. When clinical characteristics between earlier and later onset of BD were compared, subjects with early AAO had more frequent suicidal ideation/attempts, axis I comorbidity, substance use disorders, and rapid cycling course. The odds ratios associated with these variables were 1.4 (suicide ideation), 1.6 (axis I comorbidity), 1.4 (substance abuse), and 2.0 (rapid cycling course) [18].

A study with 368 patients investigated the cut-off in the age at onset in three subgroups (early, intermediate, and late). The mean age in each group was estimated to be 17.4 (SD = 2.3), 25.1 (SD = 6.2), and 40.4 years (SD = 11.3) [19].

A sample of 1,000 adults with bipolar disorder was divided into three groups: very early (<13 years), early (13–18 years), and late (>18 years) onset of mood symptoms and it resulted in 983 patients whose age at onset could be determined: 272 (27.7%) had very early onset and 370 (37.6%) experienced early onset. Early onset again was associated with higher rates of comorbid anxiety disorders and substance abuse, more recurrences, shorter periods of euthymia, greater likelihood of suicide attempts, and violence, suggesting a more severe course of disease in terms of chronicity and comorbidity [20].

An article reported the age at onset of 211 families with BD type I probands. Part of the probands was analyzed to determine the age at onset distribution. They divided the analysis into two variables: “early-onset” subgroup as ≤21 years and “late onset” subgroup as >21 years. Clinical features, such as comorbid substance abuse, rapid cycling, suicidality, and increased episode frequency, were correlated with the “early onset” group [21].

History of high depressive recurrence (without history of mania/hypomania) has been proposed as a mood subtype close to bipolar disorders. A study with 224 outpatients diagnosed with Major Depressive Disorder and with 336 outpatients with BD type II was conducted on such putative bipolar validators (early age at onset, high recurrence, mixed depression, and bipolar family history) as early age at onset of first major depressive episode (before 21 years). Early onset was the only variable which identified a major depressive disorder subgroup significantly associated with all bipolar validators. This major depressive disorder subgroup was similar to BD type II in age at onset and bipolar family history and had a high frequency of mixed depression [22].

A sample of 1,369 BD type I patients was divided into three subgroups: early onset as <22 years; intermediate onset as 25–37 years; and late onset as >40 years. The early onset subgroup had more rapid cycling, family history for affective disorders, and episodes of depression and mania when compared to other subgroups [23].

A study with two independent samples from France (*n* = 480) and from the United States (*n* = 714) was carried out with BD type I patients. Early age at onset was defined as <21 years. A correlation of early age at onset, substance use, and suicidal behavior was found [3].

### 3.2. Cannabis Use as a Risk Factor for Early Onset of BD

There is no consensus about the criteria of frequency or quantity of cannabis use in BD to establish the definition of abuse, as shown in Table 3. Criteria range from the number of joints per day in the last 12 months [8] to the qualification of “heavy consumers” (several times a day) [9]. The frequency of use was adopted as a parameter in two studies, but with different approaches. While Henquet et al. [24] stratified the cannabis use into daily, weekly, and monthly frequencies, Tijssen et al. [25] classified, in their sample, all patients who had made the use of cannabis five times or more in their lifetime.

Table 3 shows the criteria adopted in several studies to establish the frequency of cannabis use. The following variables are described: year of publication, study design, follow-up period, measures, comparisons, and frequency of cannabis use. Sample sizes ranged from 151 to 4,815.

A study with a sample of 4,815 individuals between 18 and 64 years old was examined, using the Composite International Diagnostic Interview, and it was found that cannabis use can trigger manic symptoms independently of age, ethnicity, neuroticism, sex, educational level, marital status, use of other drugs or alcohol. Frequency of use increases the risk. Patients who used it 3-4 days per week were more likely to manifest manic symptoms than those who used it less frequently [24].

A study in a prospective cohort of 705 adolescents followed up during 8 years analyzed the association between risk factors, such as cannabis use, and the manifestation of manic or depressive symptoms in bipolar patients and found that cannabis use was involved with manic manifestation [25].

A sample with 766 patients between 16 and 65 years old, 676 of them diagnosed with schizophrenia and 90 with BD, was interviewed and cannabis use was linked to early age at onset of schizophrenia and BD [9]. Another study investigated 151 patients with BD types I and II who were in psychiatric treatment and it was observed that excessive cannabis use was related to early onset of BD and that it had been used at an earlier age than the excessive use of alcohol [26].

Data from the National Epidemiological Survey of Alcohol and Related Conditions (NESARC Wave 1, 2001-2002) were analyzed and they showed that 1,905 patients were diagnosed with bipolar disorder and 7.2% of these were identified with cannabis use disorder (CUD). The prevalence of CUD in the general population is 1.2%. It was observed that the cooccurrence of BD and CUD was a risk factor for alcohol, nicotine, and other drug dependence [8].

In a study involving approximately 2,000 individuals (471 bipolar individuals and 1,761 controls), bipolar patients were 6.8 times more likely to report cannabis use during their lifetime. Almost 30% of the BD group that had made use of cannabis at least once fulfilled the criteria for CUD of DSM-IV. Individuals with BD and CUD were 1.8 times more likely to have disability due to the disorder than those with the diagnosis of BD that had no history of CUD, even after controlling sociodemographic variables, substance use, and psychiatric covariates. Individuals with BD and CUD had more mixed episodes and a higher probability of suicide attempts than those with BD but without CUD [37].

In a study selecting adult patients from France and the United States that met DSM–IV criteria for BD type I, the age at onset was classified into two subgroups: early age at onset (<21 years) and late age at onset (≥21 years). They analyzed the association of clinical and demographic variables with age at onset and polarity. The relationships between “age at onset,” “alcohol/drug misuse,” and “suicidal behavior” are the most important in both samples: early age at onset was associated with suicidal behavior (France: OR = 2.16 95% CI (1.48–3.15) Sensibility = 35%, Specificity = 46%; USA: OR = 2.05 95% CI (1.44–2.92) Sensibility = 27%, Specificity = 54%) and lifetime cannabis misuse (France: OR = 2.60 95% CI (1.51–4.48) Sensibility = 9%, Specificity = 79%; USA: OR = 1.75 95% CI (1.02–3.01) Sensibility = 30%, Specificity = 59%) [3].

A cross-sectional study was conducted on a population-based national representative sample, the National Epidemiological Survey of Alcohol and Related Conditions (NESARC). Individuals with lifetime prevalence of BD (*n* = 1, 905) were analyzed regarding sociodemographic characteristics and prevalence of comorbid psychiatric disorder among BD patients with and without CUD (in the last 12 months). They found among BD patients with CUD (*n* = 119) an earlier age at onset of the first manic or hypomanic episode (mean age = 19.5 years) and of the first depressive episode (mean age = 18.5 years) compared to patients without CUD (*n* = 1, 786), whose mean ages were 25.1 years and 24.4 years, respectively (*P* < 0.0001). In this study, CUD was associated with earlier onset of bipolar disorder and greater number of depressive and hypomanic/manic episodes [8].

A secondary analysis of data collected by the Netherlands Mental Health Survey and Incidence Study (NEMESIS)—a longitudinal study with three measurements at 1996, 1997, and 1999 (follow-up)—of the Dutch adult population (18–64 years) was conducted. At baseline, 7,076 individuals were interviewed, and, in the last follow-up, 4,848 participants were interviewed. To investigate if cannabis use predicted the first episodes of mood and anxiety disorders, the analyses were made with 3,881 people who had had no lifetime mood disorder and with 3,854 people who had had no lifetime anxiety disorder. It was found that cannabis was a predictor of subsequent mood episodes, especially bipolar disorder, and that cannabis use was associated with increasing the risk of BD (OR = 7.6; *P* < 0.001) [38].

A paper reported substantial evidence for phenotypic and genetic overlap between schizophrenia and BD. An earlier onset was found in patients that used cannabis (676 schizophrenia patients and 90 BD patients). The reduction was 9 years in the age at onset in the bipolar group [9].

In a sample of 151 BD types I and II, cannabis use was associated with an earlier onset of BD independently of the history of psychosis or polarity of the first episode [26], not only manic or psychotic, as described by Henquet et al. [24] and Öngür et al. [39]. It means that cannabis increases bipolar disorder prevalence in general. Öngür et al. (2009) reported that comorbid lifetime cannabis, but not alcohol, abuse/dependence was associated with a statistically significant 3-year-earlier age at onset of psychosis in schizophrenia (*n* = 80), schizoaffective disorder (*n* = 61), and bipolar disorder with psychotic features (*n* = 92). Patients fulfilled the criteria for cannabis abuse/dependence an additional 3 years before psychosis [39].

In a study with 324 BD types I and II patients, an independent dose-response was shown between cannabis use and age at onset of BD: there was a statistically significant decrease in age at onset with increasing levels of lifetime cannabis use, from 23.2 years (±9.7) for patients who never used cannabis or used cannabis <10 times during one month lifetime, 20.5 years (±7.3) for patients who used cannabis >10 times during one month lifetime, and 18.6 years (±5.0) for patients with a lifetime cannabis use disorder (abuse or dependence) [7].

### 3.3. Suicidal Behavior and Cannabis Use in Bipolar Disorder

According to the World Health Organization (WHO), about 3,000 people commit suicide every day worldwide—one every 40 seconds. For each suicide, 20 or more attempts are committed. The annual number of suicides is currently around one million and represents about half of all violent deaths recorded in the world [40].

Among psychiatric disorders, BD has the highest risk of suicide, reaching rates 20–30 times higher than in the general population [14]. Approximately 56% of patients with BD who committed suicide had attempted suicide at least once in their life; and 1 in 15 bipolar patients are victims of suicide [41]. The rate that expresses suicide attempts at least once in their life in bipolar patients varies between 25% and 50%; however, 8% to 19% will commit suicide [27].

The number of suicide attempts in bipolar types I and II is a subject of controversy. One study demonstrated that bipolar type II patients have higher rates of suicide risks [41]. Another study shows not statistically significant rates in suicide risk among bipolar patients either with type I or type II in a follow-up of bipolar patients up to 18 months and in a study in Barcelona that analyzes 290 bipolar patients [28].

More suicide attempts are committed by bipolar females than bipolar males (34–19%). However, higher rates of completed suicide are observed among bipolar males. Early onset of psychiatric disorders and personality disorders contributes to the risk of suicide. BD associated with substance use in males doubles the number of suicidal behaviors [16].

The early age at onset of BD and gender, prior suicide attempts, suicide in different mood episodes of bipolar disorder, episode polarity and polarity of first affective episode, and drug and alcohol abuse disorders show a significantly increased risk of suicide attempts [41]. The early onset of this disorder increases the risk of suicide and that early onset is around age 20 [42, 43].

Based on a cohort of 6,086 (60% females) patients followed up annually 2005–2012, it was found that early onset is a significant predictor of suicide attempts only in females. Clinical data was extracted from Swedish National Quality Register for Bipolar Disorder [16]. Early age at onset was also associated with suicide attempts in samples from France and the United States [3].

Another study with a population-based longitudinal cohort sample included 1,542 patients with bipolar depression attending a registry of 388,624 inhabitants of Keelung City, Taiwan, from 1999 to 2004. This cohort was followed until the end of 2008 and data were from the National Health Insurance Dataset (NHID) and the National Mortality Registry (NMR). The risk of suicide in these patients was doubled in comparison with other depressive patients [44].

Table 4 shows studies associating suicidal behavior and BD. The following variables are described: year of publication; number of individuals; suicide attempts; suicide attempts and substance use; suicide attempts; and cannabis use. Sample sizes ranged from 170 to 31,000. The following percentages in bipolar patients were found: suicide attempts 3.6–42%; suicide attempts and substance use ranging from 5 to 60%; and suicide attempts and cannabis use ranging from 15 to 42%. It is important to notice that higher percentages of suicide attempts were found in the studies with smaller number of participants, which is statistically understandable, but the real number of patients that attempted suicide was significant in all studies taking in credit that all suicide attempts can potentially lead to death.

A sample of 1,556 bipolar patients was examined along 2 years of follow-up. They were participants in the Systematic Treatment Enhancement Program for Bipolar Disorder (STEP-BD), a multicenter study which evaluates longitudinal outcomes in patients with BD. This study analyzed the association between baseline clinical and demographic variables and subsequent suicide attempts and completions. Suicidal ideation, percent of anxiety, depressed and irritable days in past year, history of suicide attempts, age at onset, marital and smoking status, age, and gender were considered for comparison. The sample with complete baseline data was categorized by whether or not participants experienced a suicide attempt or completion over the 2-year follow-up period. A rate of 3.66% (57 patients) attempted or completed suicide. After analysis, only history of suicide (OR = 4.52) and percent of depressed days in the past year (OR = 1.16) were significant. The age at onset of BD (<13 years) presented OR = 1.37 [27].

A prospective study was conducted in the Jorvi Bipolar Study (JoBS) screening 1,630 patients, of whom 546 obtained a positive MDQ screen or suspicion of BD. 490 patients of this sample could be interviewed; however, 201 had the diagnosis of BD confirmed by the Structured Clinical Interview for DSM-IV Disorders (SCID-I) and 10 patients refused to participate. This research aimed to investigate the risk for suicide attempts in psychiatric inpatients and outpatients with BD, and it found that during the 18-month follow-up, 20% of patients (35/176) attempted suicide. In a Cox regression model, baseline previous suicide attempts, hopelessness, depressive phase at index episode, and younger age at intake were independent risk factors for suicide attempts during follow-up, whereas factors such as bipolar I or II or comorbidity did not reach statistical significance [28].

Recent affective episodes predicted attempted suicide during follow-up in male (OR = 3.63) and in female (OR = 2.81) patients as well as previous suicide attempts (male: OR = 3.93; female: OR = 4.24) and recent psychiatric inpatient care (male: OR = 3.57; female: OR = 2.68). Those with many lifetime depressive episodes were more likely to attempt suicide. Comorbid substance use disorder was a predictor in male, while many lifetime mixed episodes, early onset of mental disorder, personality disorder, and social problems related to the primary group were predictors in women [16].

A study was conducted with a group of 1,369 bipolar patients, divided into three subgroups: the first was represented by patients who had had an early age at onset, the second an intermediate age at onset of BD, and the third a late onset, producing a sample of 1,225 individuals (144 with borderline values were excluded). Results showed that 44.3% of the first group attempted suicide while in the second and third groups, respectively, the rate was 33.7 and 28.7%. The early onset group in comparison with the others had a greater frequency of suicide attempts [23].

In a prospective study with a sample of 2,219 bipolar patients who provided data about their lifetime history, 663 (29.9%) had made at least one suicide attempt. Factors that were associated with a history of suicide attempts included the following variables: female gender, a history of alcohol abuse, a history of substance abuse,* early onset*, longer disorder duration, greater depressive symptom severity, current benzodiazepine use, higher overall symptom severity, and poor compliance. Of the 663 patients with suicide attempts, 17.3% of them had lifetime cannabis abuse. Of the sample without suicidal behavior (1,556), 10.7% had lifetime cannabis abuse [29].

A study conducted with a bipolar cohort of 87 males and 70 females aimed to detect factors that may be predictive for suicide attempt. Among 157 patients, 59 of them had a history of at least one suicide attempt. White race, family history of completed suicide, and history of cocaine abuse/dependence were predictive of suicide attempt histories [30].

Clinical and dimensional characteristics in bipolar patients were analyzed through diagnostic interview and questionnaires. In a sample of 652 patients, 280 (42.9%) suicide attempts were detected. Some variables were associated with a lifetime history of suicidal behavior like being a woman, a history of head injury, tobacco misuse, early age at onset, high number of depressive episodes, positive history of rapid cycling, alcohol misuse, and social phobia. Data was analyzed comparing two different groups: bipolar patients with and without suicidal behavior (BD + SB and BD − SB) and an earlier age at onset was associated with suicidal behavior (23.1 ± 9.1 in BP + SB versus 27.0 ± 11.1 in BD − SD). Cannabis misuse was indicated in 41 (15.1%) of 280 (42.9%) bipolar patients that made a suicide attempt [31].

In a cohort of 3,083 bipolar patients, 140 (4.6%) had a suicide event (8 died by suicide and 132 attempted suicide). The strongest predictor of a suicide event was a history of suicide attempt, in line with prior literature. Additional predictors were younger age, a high total score on the personality disorder questionnaire, and a high percentage of days spent depressed in the year prior to study entry [32].

A meta-analysis was conducted in order to relate comorbidities and suicide attempts in patients with BD. Twenty-nine of 222 studies assessed for eligibility met the inclusion criteria, comprising a total of 31,294 individuals with BD, of whom 6,308 (20.1%) had documented suicide attempts. There were consistent findings across the studies included. As compared to controls, subjects with BD and comorbid alcohol and other substance use disorder were more likely to attempt suicide [33].

The association between suicide and substance abuse in bipolar patients can be demonstrated by substantial evidence of suicidal behavior in patients with comorbidities for substance use disorders. This relationship can be sustained on the basis of data stating that cannabis use affects negatively the course of bipolar disorder. The effects of substance use on suicidal behavior were registered by a large epidemiological study in 1,643 bipolar patients among 43,093 general-population respondents who were interviewed in the 2001-2002 by the National Epidemiologic Survey on Alcohol and Related Conditions (NESARC) [45].

The use of cannabis reduces the age at onset of BD; it may also increase manic episodes and their durations and may increase the risk of suicide in patients with bipolar depression. A study involving 101 patients concluded that the first exposure to cannabis represented strong evidence of an earlier age at onset of first depressive episode [46].

Cannabis use in patients with bipolar disorder increases the incidence of psychotic symptoms [47] and suicide attempts [33] and still decreases the response to treatment by lithium compared to patients who do not use this substance [48].

The abusive use of cannabis may act as a predictor of early onset of bipolar disorder and both conditions can interfere with increasing the risk of suicide in patients with BD [29]. Greater suicidal behavior in bipolar patients who experienced a reduction in the age at onset in the context of cannabis use was also demonstrated [37].

A group of 125 bipolar patients with psychotic features was analyzed and it was demonstrated that bipolar disorder occurred earlier in a subgroup that had substance abuse associated with other comorbidities from axis I [49].

A meta-analysis of 29 studies evaluated suicide attempts and substance use (including cannabis) specifically in BD population. It comprised 31,294 individuals with BD of which 6,308 (20.1%) had suicide attempts. A consistent association of alcohol and drug use with suicide attempts was found. Cannabis use disorders were evaluated in 4 studies comprising 3,439 individuals with specific information on BD, suicide attempts, and CUD (559 with BD and lifetime cannabis use disorders). Individuals misusing cannabis showed an increased risk of suicide (OR = 1.44) when compared with their noncomorbid counterparts [33].

## 4. Discussion

### 4.1. Age at Onset of Bipolar Disorder

The definition of early age at onset of BD is not clear; however, it is associated with poor prognosis of BD [23]. If an early diagnosis of BD is not established, the treatment starts late, compromising a good outcome [34]. Nine studies between 2000 and 2012 were evaluated and a wide variation in the meaning of “early age at onset” was found (6–22 years), as shown in Table 2.

The definition of the age at onset of bipolar disorder is important because there is robust evidence suggesting that the age at onset distribution of individuals affected with bipolar disorder is composed of at least two age distributions (early and late onset) [10, 17, 18, 22, 43], three divided into early, intermediate, and late [19, 21, 23] and only one into very early, early, and late onset [20]. The three normal distributions (early, intermediate, and late onset) may reflect genetic heterogeneity within bipolar I disorder [23].

Results may vary in the literature relating to age at onset because different criteria were used to establish the definition of age at onset. Hamshere et al. [23] incorporated the duration of the disorder at the time of the interview and gender as covariates, while Benazzi and Akiskal [22] used age at assessment-time, gender, bipolar type II, and mania/hypomania family history. Perlis et al. [20] also included the duration of the disorder and finally Lin et al. [21] included the variables for age of contact and gender.

The definition of very early age at onset is even more troublesome because the first symptoms of mood variation that could be diagnosed as the first bipolar episode are difficult to diagnose by MINI and other instruments [20].

The relationship between early age at onset and the severity of bipolar disorder is well established. Early age at onset is associated with a greater number of rapid cycling [18, 21, 23], mixed episodes [10], and psychotic episodes [10, 17] and panic disorder [10], anxiety disorder [18, 20], substance use disorder [3, 18, 20, 21], and major depression [22], even worse response to lithium [10] and suicidal behavior [3, 18, 20, 21].

### 4.2. Early Age at Onset of Bipolar Disorder and Cannabis Use

Cannabis use is a risk factor for early age at onset of BD. Substance use disorder is associated with a worse course of BD. Cannabis is the most common illicit substance used among individuals with BD (7.2% in BD* versus* 1.2% in general population). The use of marijuana seems to be a predictor of mood episodes in BD patients and it reduces the age at onset in 6–9 years [8, 9].

Cannabis use disorder triggers earlier episodes of BD when compared with alcohol use disorder [50]. Especially in cases of cannabis abuse, the relationship between abuse and age at onset is not well understood. It happens because substance use disorder can be a prodromal symptom of mood disorder and can be both a consequence and a cause of BD [30]. The interaction of childhood stressor events and cannabis use appears to be the potential factor for a vicious cycle [51, 52]. Another interesting point was that patients with lifetime cannabis and alcohol abuse/dependence had a later onset compared with those who had only cannabis abuse/dependence [39].

### 4.3. Cannabis Use and Suicide Behavior in Bipolar Disorder

There is an association between early age at onset of BD and suicide attempts. Among psychiatric disorders, BD has the highest risk of suicide [14]. Early age at onset is associated with suicide attempts [3] and is considered a predictor, especially in females [16]. It is still necessary to elucidate whether early age at onset in BD is an independent risk factor for suicide attempts or whether it depends on other factors, such as disorder severity, rapid cycling, more psychiatric comorbidity, abuse in childhood, family history of mood disorder, differences of gender, and BD subtypes [53, 54]. The difference in suicide rates between bipolar types I and II is controversial. One study suggests higher suicide rates in BD type II group [41]. But when gender was compared, females showed higher rate of suicide attempts, while males presented higher rates of completed suicide [16]. There is no enough data to differentiate between suicide rates in bipolar types. BD type II was associated with higher rates of suicide attempts [41], while other studies have shown no significant differences between BD types I and II [28].

Cannabis use in patients with BD increases the risk of suicide attempts [33]. The findings of an association between cannabis use, early age at onset, and suicide attempts may be taken as a support of the view that cannabis negatively affects the course of the disorder [46]. Substance use disorder may be genetically associated with bipolar disorder. Recent research confirms the importance of the genetics of the bipolar disorder, although the involvement of no specific chromosome region or gene has been specifically confirmed [55–58].

There is literature evidence that BD and alcohol dependence are highly genetically influenced. The pathophysiology of this interaction is not completely elucidated and thus points to the importance of researching common biomarkers of both disorders for a better understanding of the course of the disorder. It may be hypothesized that cannabis use and BD may share a common genetic background.

### 4.4. Neurophysiological Changes Associated with Cannabis Use

The neuropathological bases of comorbidity between bipolar disorder and cannabis use disorder are a challenge because there are few studies addressing the pathophysiology of these two disorders. Areas of medial temporal cortex and prefrontal and subcortical regions with structural and functional abnormalities are associated with emotional and motivational processing in adolescents with BD [59] and substance use disorder [60]. Thus, neurophysiological dysfunctions may overlap in some individuals predisposed to the development of both disorders: BD and substance use disorder [61].

Only one study addressed the relationship between bipolar patients and the expression of CB1-R and did not find a direct relation to neither increase nor decrease the density in bipolar patients. However, it was observed that bipolar patients who were taking first-generation antipsychotics had their levels of CB1-R immunoreactive glial cells reduced [37]. Type 1 cannabinoid receptor, CB1-R, is a G protein-coupled receptor and located mainly in the central and peripheral nervous system. It is activated by the compound THC found in cannabis. CB1-R is related to search behavior in patients with cannabis abuse. This is due to influence on the mesolimbic pathway, especially in the nucleus accumbens region [62]. Another research found that polymorphism of AKT1 gene may be related to the use of cannabis and to the developing of psychosis, but not specifically for bipolar patients [63].

Bipolar adolescents with cooccurring cannabis use disorder had structural differences in frontal and temporal cortical regions and the right caudate nucleus, which is extended and is related to emotional and motivational regulation [61]. In addition, patients who use cannabis and tobacco had lower activation of the right hippocampus when compared with controls [64]. In patients with cooccurrence of BD and cannabis use disorder an increase in gray matter volume in the right caudate and precentral gyrus and increased gray matter density in the occipital, middle right fusiform, and cerebellar vermis were observed, while there was a reduction in gray matter volume in the left fusiform [61].

Abnormalities in the subcortical region have been linked to dysfunction in the reward system in patients with substance use disorder. The activity of the caudate is related to desire [65] and the fusiform gyrus, which is altered in bipolar patients with cannabis use disorder, is associated with craving and drug seeking behavior [66]. The results of studies attempting to correlate structural and functional changes in the brains of patients with BD and cannabis use disorder found some limitations because they were not able to differentiate preexisting structural brain abnormalities from the consequences of repeated exposure to abused substances [67]. Furthermore, a preliminary study with a small sample of patients (*n* = 10) found no significant changes in the brain of patients who were frequently using cannabis [68].

Several studies have demonstrated that BD is more prevalent in individuals who have experienced early-life events stressors. Neuroendocrine, autonomic, immune, and oxidative responses are triggered, once a state of chronic inflammation is elicited, altering cellular mediators of plasticity and energy metabolism, besides the deleterious programing of the hypothalamic-pituitary-adrenal (HPA) axis [50]. van Leeuwen et al. [51] proposed opposite effects on the hypothalamic-pituitary-adrenal (HPA) axis when childhood abuse and cannabis use cooccur. That is why cannabis seems to decrease the programming of HPA while childhood trauma is linked as a stressor to HPA, triggering a factor for long-term hyperactivation of HPA [52].

The catechol-O-methyltransferase has been studied because it is involved in the metabolism of catecholamines (dopamine, adrenaline, and noradrenaline). Massat et al. [69] observed the influence of catechol-O-methyltransferase variants on major depression and BD, particularly in early onset subjects. Experimental studies support a theory which shows an interaction between cannabis and catechol-O-methyltransferase as a mechanism for cognitive abnormalities [9].

A reduced expression of polyamines was observed in bipolar patients with completed suicide. The polymorphism of the gene responsible for enzyme SAT1 is related to dysfunction in the catabolism of polyamines [70]. Bipolar patients who had been hospitalized with or without suicidal ideation had a reduced amount of SAT1 when compared with those who had not been hospitalized [71].

### 4.5. Limitations

This study has some limitations. There is sparse literature on the issue involving cannabis use, early age at onset of BD, and suicide attempts. The definition of early age at onset is not consensual; therefore studies may not be entirely comparable. Some studies report data on the bipolar group as a whole, not specifying the relationship of cannabis use, early age at onset, and suicide attempts. Other studies report data involving the early age at onset in bipolar and schizophrenia making it harder to distinguish to which group of patients the study is referring. A definition of what constitutes a prodromal phase or an index episode in many studies is lacking. Information on whether there is only cannabis use/abuse or other psychiatric comorbidities in terms of drug use/abuse is missing in many studies. The definition of cannabis use/abuse is subject of controversy. There are arbitrary criteria for specifying use and abuse. Finally, some studies published data only on BD type I and others on BD type II and both (BD I and II) and, in some of them, it is not specified whether the sample refers to BD and schizophrenia [72–75].

However, in this review, as in other studies, the relationship between abuse/dependence and age at onset differentiated on the basis of psychiatric diagnosis or gender aspects has not been investigated, although some of these associations are potentially plausible.

## 5. Conclusion

In conclusion, the age at onset of bipolar disorder was reviewed due to its prognostic value, making it clear that the first episode should have an early diagnosis. The evidence of cannabis use as a risk factor to early onset of bipolar disorder highlights the necessity to be attentive to the first bipolar episode so that treatment can be started promptly. The use of cannabis is an important factor that may trigger early onset of BD and, by itself, is associated with higher rates of suicidal behavior in BD. However, it is not clear whether the effect of cannabis at the age at onset and suicide attempts are independent of each other or not.

## Figures and Tables

**Figure 1 fig1:**
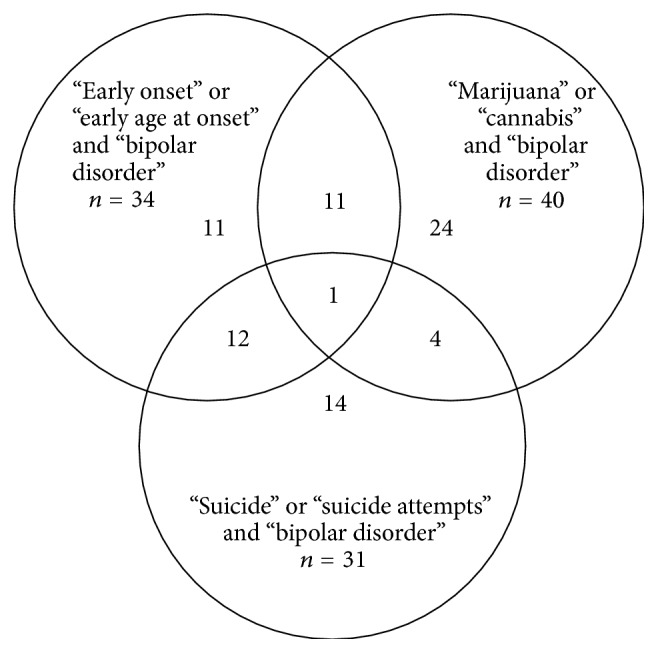


**Table 1:** Flowchart—selection of articles (see Figure 1).

Initial search					
“Early age at onset” and “bipolar disorder”	“Early onset” and “bipolar disorder”	“Suicide attempts” and “bipolar disorder”	“Suicide” and “bipolar disorder”	“Marijuana” and “bipolar disorder”	“Cannabis” and “bipolar disorder”
626	1,017	1,069	2,289	190	143

Inclusion of the articles fulfilling the entry criteria^*^ (at least one)					
“Early age at onset” or “early onset” and “bipolar disorder”		“Suicide attempts” or “suicide” and “bipolar disorder”		“Marijuana” or “cannabis” and “bipolar disorder”	

51		44		59	

Exclusion of repetitive and duplicate articles					

17		13		19	

Final analysis					

34		31		40	

^*^(1) The selected study should provide data that allowed evaluating the age at onset of bipolar disorder.

(2) The paper should present the proportion of bipolar patients who used cannabis.

(3) Patients included in these studies should have had an early age at onset of BD or suicide attempts.

**Table 2:** Definitions of age at onset of bipolar disorder.

Study	Design	Sample (bipolar patients)^a^	Very early age at onset (in years)	Early age at onset (in years)	Intermediate age at onset (in years)	Late age at onset (in years)
Schürhoff et al., 2000^b^ [10]	Cross-sectional	210	—	<18	—	>40

Schulze et al., 2002^b^ [17]	Cross-sectional	169	—	≤20	—	≥35

Post et al., 2003^b^ [18]	Cross-sectional	320 (202 female; 118 male)	—	≤18	—	>18

Bellivier et al., 2003^c^ [19]	Cross-sectional	368	—	Median age: 17.4	Median age: 25.1	Median age: 40.4

Perlis et al., 2004^c^ [20]	Cross-sectional	1,000	<13	13–18	—	>18

Lin et al., 2006^c^ [21]	Cross-sectional	1,856 (211 probands)	—	≤21 Mean age: 16.6	22–28 Mean age: 26	>28 Mean age: 34.7

Benazzi and Akiskal, 2008 [22]	Cross-sectional	560^d^	—	<21	—	>21

Hamshere et al., 2009^c^ [23]	Cross-sectional	1,369	—	Limit: <22 mean age: 18.7 ± 3.7	Limit: 25–37 mean age: 28.3 ± 5.5	Limit: >40 mean age: 43.3 ± 9.1

Etain et al., 2012^c^ [3]	Cross-sectional	652	—	<21	—	≥21

(based on Hamshere et al. 2009 [23]).

^a^Some studies do not classify the patients whose diagnosis was included.

^b^Age of both bipolar disorder (BD) type I and BD type II.

^c^Age of BD type I.

^d^336 BD type II and 224 unipolar major depressive disorder.

**Table 3:** Frequency of use of cannabis in bipolar disorder.

Study	Design	Sample (*n*)	Follow-up (in years)	Measures	Comparisons	Frequency of cannabis use
Henquet et al., 2006 [24]	Cohort	4,815 individuals^a^ 18–64 years	3	Composite International Diagnostic Interview (CIDI)	The baseline cannabis use was assessed with the occurrence of mania in the follow-up	Less than once a month; 1–3 days/month; 1-2 days/week; 3-4 days/week and nearly every day.

Tijssen et al., 2010 [25]	Cohort	705 patients 14–24 years	8	Munich-Compo site International Diagnostic Interview (M-CIDI)	The onset of manic/depressive symptoms was assessed with the following risk factors (a family history of mood disorders, trauma, substance use, attention-deficit/hyperactivity disorder (ADHD), and temperamental/personality traits)	Lifetime cannabis use was considered in case they reported at baseline that they had used cannabis five times or more

de Hert et al., 2011 [7]	Cross- sectional	766 patients (676 with schizophrenia and 90 with bipolar disorder) 16–65 years	—	Composite International Diagnostic Interview (CIDI), Clinical Global Impression (CGI), and Global Assessment of Functioning (GAF)	A linear regression between the age at onset was done considering the following variables: cannabis use, diagnosis, and gender	Used CIDI (Composite International Diagnostic Interview) for lifetime substance use and classified patients as “heavy users” when consumption was several times a day.

Lagerberg et al., 2011 [26]	Cross-sectional	151 bipolar patients (91 BD I and 60 BD II)	—	Clinical assessments carried out by trained clinical psychologists and psychiatrists	The bivariate analyses revealed significant correlations between age at onset and gender, age, BD type, excessive cannabis use, and sequencing	Patients who met DSM-IV criteria for substance use disorder or had predominant weekly use of cannabis for a period of 4 years from 11–15 years, 16–20 years, 21–27 years, 28–44 years, 45–60 years, and 60 years and more were considered “excessive cannabis use”

LevRan et al., 2013 [9]	Cross-sectional	1,905 bipolar individuals	—	Alcohol use disorder and associated disabilities interview schedule	Rates of CUD in the past 12 months were 7.2%, compared to 1.2% in the general population. Logistic regression models adjusting for sociodemographic variables indicated that cooccurring CUD was at increased risk for nicotine dependence, alcohol and drug use disorders, and antisocial personality disorder compared to those without CUD.	Number of joints consumed with the number of days when cannabis was used in the last 12 months. Frequency was defined as ranging from “almost daily” to “once a year.”

^a^The sample was found to be representative of the Dutch population in terms of gender, marital status and level of urbanisation, with the exception of a slight under-representation of individuals in the age group 18–24 years.

**Table 4:** Bipolar disorder and suicide attempts.

Study	Design	Sample (*n*)	Follow-up time (in years)	Suicides in BD	Suicide attempts in BD and substance use	Suicide attempts in BD and cannabis
Marangell et al., 2006 [27]	Cohort	1,556	2	3.6% (*n* = 57) suicide attempts (*n* = 50) or completions (*n* = 7)	—^a^	—^a^

Valtonen et al., 2006 [28]	Cohort	176	1.5	20% (*n* = 35) attempts, 1% (*n* = 2) completions	45% alcohol, 46% smoking	—^a^

Tidemalm et al., 2014 [16]	Cohort	6,086 (male = 2,408 female = 3,678)^b^	After attempted suicide, ranging from 19–30	Male: 4,1% (*n* = 98) Female: 6,8% (*n* = 253)	—^a^	—^a^

Hamshere et. al., 2009 [23]	Cross-sectional	1,369	—^a^	Early onset (44.3%) (*n* = 235) Mid-onset (33.7%) (*n* = 129) Late onset (28.7%) (*n* = 31) Suicide attempts	—^a^	—^a^

Bellivier et al., 2011 [29]	Prospective observational	2,219	2	29.9%	—^a^	17.3%

Cassidy^*^, 2011 [30]	Cohort	157	—^a^	37.6%	Nicotine: 66.2% Alcohol: 36.3% Cocaine: 23.6% Benzodiazepine: 5.7% Amphetamine: 7.6% Opiate: 5.1% Hallucinogen: 9.6%	42.7%

Parmentier et al., 2012 [31]	Cross-sectional	652	—^a^	42.9%	—^a^	15.1%

Antypa et al., 2013 [32]	Cohort	3,083	—^a^	4.6%	—^a^	—^a^

Carrà et al., 2014 [33]	Meta-analysis	31,294	—^a^	—^a^	20.1%	—^a^

^*^Cassidy: the rates of substance use and cannabis use are associated with the total sample. It does not represent necessarily interaction with the rate of suicide attempts.

^a^No data available.

^b^Including Bipolar Disorder Subtypes 1 and 2, Unspecified type and Schizoaffective Disorder of Bipolar Type.

## References

[1] Gao K., Tolliver B. K., Kemp D. E. (2008). Differential interactions between comorbid anxiety disorders and substance use disorder in rapid cycling bipolar I or II disorder. *Journal of Affective Disorders*.

[2] Cerullo M. A., Strakowski S. M. (2007). The prevalence and significance of substance use disorders in bipolar type I and II disorder. *Substance Abuse: Treatment, Prevention, and Policy*.

[3] Etain B., Lajnef M., Bellivier F. (2012). Clinical expression of bipolar disorder type I as a function of age and polarity at onset: convergent findings in samples from France and the United States. *Journal of Clinical Psychiatry*.

[4] Dell'Osso B., Buoli M., Bortolussi S., Camuri G., Vecchi V., Altamura A. C. (2011). Patterns of Axis i comorbidity in relation to age in patients with Bipolar Disorder: a cross-sectional analysis. *Journal of Affective Disorders*.

[5] van Rossum I., Boomsma M., Tenback D., Reed C., van Os J. (2009). Does cannabis use affect treatment outcome in bipolar disorder? A longitudinal analysis. *Journal of Nervous and Mental Disease*.

[6] Strakowski S. M., Sax K. W., McElroy S. L., Keck P. E., Hawkins J. M., West S. A. (1998). Course of psychiatric and substance abuse syndromes co-occurring with bipolar disorder after a first psychiatric hospitalization. *Journal of Clinical Psychiatry*.

[7] de Hert M., Wampers M., Jendricko T. (2011). Effects of cannabis use on age at onset in schizophrenia and bipolar disorder. *Schizophrenia Research*.

[8] Lagerberg T. V., Kvitland L. R., Aminoff S. R. (2014). Indications of a dose-response relationship between cannabis use and age at onset in bipolar disorder. *Psychiatry Research*.

[9] Lev-Ran S., le Foll B., McKenzie K., George T. P., Rehm J. (2013). Bipolar disorder and co-occurring cannabis use disorders: characteristics, co-morbidities and clinical correlates. *Psychiatry Research*.

[10] Schürhoff F., Bellivier F., Jouvent R. (2000). Early and late onset bipolar disorders: two different forms of manic-depressive illness?. *Journal of Affective Disorders*.

[11] Lish J. D., Dime-Meenan S., Whybrow P. C., Price R. A., Hirschfeld R. M. A. (1994). The National Depressive and Manic-depressive Association (DMDA) survey of bipolar members. *Journal of Affective Disorders*.

[12] Hawton K., Sutton L., Haw C., Sinclair J., Harriss L. (2005). Suicide and attempted suicide in bipolar disorder: a systematic review of risk factors. *Journal of Clinical Psychiatry*.

[13] Anderson I. M., Haddad P. M., Scott J. (2012). Bipolar disorder. *British Medical Journal*.

[14] Pompili M., Gonda X., Serafini G. (2013). Epidemiology of suicide in bipolar disorders: a systematic review of the literature. *Bipolar disorders*.

[15] Ösby U., Brandt L., Correia N., Ekbom A., Sparén P. (2001). Excess mortality in bipolar and unipolar disorder in Sweden. *Archives of General Psychiatry*.

[16] Tidemalm D., Haglund A., Karanti A., Landén M., Runeson B. (2014). Attempted suicide in bipolar disorder: risk factors in a cohort of 6086 patients. *PLoS ONE*.

[17] Schulze T. G., Müller D. J., Krauss H. (2002). Further evidence for age of onset being an indicator for severity in bipolar disorder. *Journal of Affective Disorders*.

[18] Post R. M., Leverich G. S., Kupka R. W. (2003). Early age at onset as a risk factor for poor outcome of bipolar disorder. *Journal of Psychiatric Research*.

[19] Bellivier F., Golmard J. L., Rietschel M. (2003). Age at onset in bipolar I affective disorder: further evidence for three subgroups. *The American Journal of Psychiatry*.

[20] Perlis R. H., Miyahara S., Marangell L. B. (2004). Long-term implications of early onset in bipolar disorder: data from the first 1000 participants in the systematic treatment enhancement program for bipolar disorder (STEP-BD). *Biological Psychiatry*.

[21] Lin P.-I., McInnis M. G., Potash J. B. (2006). Clinical correlates and familial aggregation of age at onset in bipolar disorder. *American Journal of Psychiatry*.

[22] Benazzi F., Akiskal H. S. (2008). How best to identify a bipolar-related subtype among major depressive patients without spontaneous hypomania: superiority of age at onset criterion over recurrence and polarity?. *Journal of Affective Disorders*.

[23] Hamshere M. L., Gordon-Smith K., Forty L. (2009). Age-at-onset in bipolar-I disorder: mixture analysis of 1369 cases identifies three distinct clinical sub-groups. *Journal of Affective Disorders*.

[24] Henquet C., Krabbendam L., de Graaf R., ten Have M., van Os J. (2006). Cannabis use and expression of mania in the general population. *Journal of Affective Disorders*.

[25] Tijssen M. J. A., van Os J., Wittchen H. U., Lieb R., Beesdo K., Wichers M. (2010). Risk factors predicting onset and persistence of subthreshold expression of bipolar psychopathology among youth from the community. *Acta Psychiatrica Scandinavica*.

[26] Lagerberg T. V., Sundet K., Aminoff S. R. (2011). Excessive cannabis use is associated with earlier age at onset in bipolar disorder. *European Archives of Psychiatry and Clinical Neuroscience*.

[27] Marangell L. B., Bauer M. S., Dennehy E. B. (2006). Prospective predictors of suicide and suicide attempts in 1,556 patients with bipolar disorders followed for up to 2 years. *Bipolar Disorders*.

[28] Valtonen H. M., Suominen K., Mantere O., Leppämäki S., Arvilommi P., Isometsä E. T. (2006). Prospective study of risk factors for attempted suicide among patients with bipolar disorder. *Bipolar Disorders*.

[29] Bellivier F., Yon L., Luquiens A. (2011). Suicidal attempts in bipolar disorder: results from an observational study (EMBLEM). *Bipolar Disorders*.

[30] Cassidy F. (2011). Risk factors of attempted suicide in bipolar disorder. *Suicide and Life-Threatening Behavior*.

[31] Parmentier C., Etain B., Yon L. (2012). Clinical and dimensional characteristics of euthymic bipolar patients with or without suicidal behavior. *European Psychiatry*.

[32] Antypa N., Antonioli M., Serretti A. (2013). Clinical, psychological and environmental predictors of prospective suicide events in patients with Bipolar Disorder. *Journal of Psychiatric Research*.

[33] Carrà G., Bartoli F., Crocamo C. (2014). Attempted suicide in people with co-occurring bipolar and substance use disorders: systematic review and meta-analysis. *Journal of Affective Disorders*.

[34] Birmaher B., Axelson D., Strober M. (2009). Comparison of manic and depressive symptoms between children and adolescents with bipolar spectrum disorders. *Bipolar Disorders*.

[35] Potash J. B., Toolan J., Steele J. (2007). The bipolar disorder phenome database: a resource for genetic studies. *The American Journal of Psychiatry*.

[36] Merikangas K. R., Jin R., He J.-P. (2011). Prevalence and correlates of bipolar spectrum disorder in the World Mental Health Survey Initiative. *Archives of General Psychiatry*.

[37] Agrawal A., Nurnberger J. I., Lynskey M. T. (2011). Cannabis involvement in individuals with bipolar disorder. *Psychiatry Research*.

[38] van Laar M., van Dorsselaer S., Monshouwer K., de Graaf R. (2007). Does cannabis use predict the first incidence of mood and anxiety disorders in the adult population?. *Addiction*.

[39] Öngür D., Lin L., Cohen B. M. (2009). Clinical characteristics influencing age at onset in psychotic disorders. *Comprehensive Psychiatry*.

[40] World Health Organization (2008). *Preventing Suicide: A Global Imperative*.

[41] Rihmer Z., Kiss K. (2002). Bipolar disorders and suicidal behaviour. *Bipolar Disorders*.

[42] Song J. Y., Yu H. Y., Kim S. H. (2012). Assessment of risk factors related to suicide attempts in patients with bipolar disorder. *Journal of Nervous and Mental Disease*.

[43] Angst F., Stassen H. H., Clayton P. J., Angst J. (2002). Mortality of patients with mood disorders: follow-up over 34–38 years. *Journal of Affective Disorders*.

[44] Chang J.-C., Chen H.-H., Yen A. M.-F., Chen S. L.-S., Lee C.-S. (2012). Survival of bipolar depression, other type of depression and comorbid ailments: ten-year longitudinal follow-up of 10,922 Taiwanese patients with depressive disorders (KCIS no. PSY1). *Journal of Psychiatric Research*.

[45] Oquendo M. A., Currier D., Liu S.-M., Hasin D. S., Grant B. F., Blanco C. (2010). Increased risk for suicidal behavior in comorbid bipolar disorder and alcohol use disorders: results from the National Epidemiologic Survey on Alcohol and Related Conditions (NESARC). *Journal of Clinical Psychiatry*.

[46] Kvitland L. R., Melle I., Aminoff S. R., Lagerberg T. V., Andreassen O. A., Ringen P. A. (2014). Cannabis use in first-treatment bipolar I disorder: relations to clinical characteristics. *Early Intervention in Psychiatry*.

[47] Katz G., Durst R., Shufman E., Bar-Hamburger R., Grunhaus L. (2010). Cannabis abuse and severity of psychotic and affective disorders in Israeli psychiatric inpatients. *Comprehensive Psychiatry*.

[48] Goldberg J. F., Garno J. L., Leon A. C., Kocsis J. H., Portera L. (1999). A history of substance abuse complicates remission from acute mania in bipolar disorder. *Journal of Clinical Psychiatry*.

[49] Pini S., Dell'Osso L., Mastrocinque C. (1999). Axis I comorbidity in bipolar disorder with psychotic features. *British Journal of Psychiatry*.

[50] Walker A. J., Kim Y., Blair Price J. (2014). Stress, inflammation, and cellular vulnerability during early stages of affective disorders: biomarker strategies and opportunities for prevention and intervention. *Frontiers in Psychiatry*.

[51] van Leeuwen A. P., Creemers H. E., Greaves-Lord K., Verhulst F. C., Ormel J., Huizink A. C. (2011). Hypothalamic-pituitary-adrenal axis reactivity to social stress and adolescent cannabis use: the TRAILS study. *Addiction*.

[52] Heim C., Newport D. J., Mletzko T., Miller A. H., Nemeroff C. B. (2008). The link between childhood trauma and depression: insights from HPA axis studies in humans. *Psychoneuroendocrinology*.

[53] Leboyer M., Henry C., Paillere-Martinot M.-L., Bellivier F. (2005). Age at onset in bipolar affective disorders: a review. *Bipolar Disorders*.

[54] Garno J. L., Goldberg J. F., Ramirez P. M., Ritzler B. A. (2005). Impact of childhood abuse on the clinical course of bipolar disorder. *British Journal of Psychiatry*.

[55] Craddock N., Sklar P. (2009). Genetics of bipolar disorder: successful start to a long journey. *Trends in Genetics*.

[56] Farmer A., Elkin A., McGuffin P. (2007). The genetics of bipolar affective disorder. *Current Opinion in Psychiatry*.

[57] Serretti A., Mandelli L. (2008). The genetics of bipolar disorder: genome ‘hot regions,’ genes, new potential candidates and future directions. *Molecular Psychiatry*.

[58] Psychiatric GWAS Consortium Coordinating Committee, Cichon S., Craddock N. (2009). Genomewide association studies: history, rationale, and prospects for psychiatric disorders. *The American Journal of Psychiatry*.

[59] DelBello M. P., Adler C. M., Strakowski S. M. (2006). The neurophysiology of childhood and adolescent bipolar disorder. *CNS Spectrums*.

[60] Hyman S. E., Malenka R. C., Nestler E. J. (2006). Neural mechanisms of addiction: the role of reward-related learning and memory. *Annual Review of Neuroscience*.

[61] Jarvis K., DelBello M. P., Mills N., Elman I., Strakowski S. M., Adler C. M. (2008). Neuroanatomic comparison of bipolar adolescents with and without cannabis use disorders. *Journal of Child and Adolescent Psychopharmacology*.

[62] Melis M., Pistis M. (2012). Hub and switches: endocannabinoid signalling in midbrain dopamine neurons. *Philosophical Transactions of the Royal Society B: Biological Sciences*.

[63] Uher R. (2014). Gene-environment interactions in severe mental illness. *Frontiers in Psychiatry*.

[64] Jacobsen L. K., Mencl W. E., Westerveld M., Pugh K. R. (2004). Impact of cannabis use on brain function in adolescents. *Annals of the New York Academy of Sciences*.

[65] Miller N. S., Goldsmith R. J. (2001). Craving for alcohol and drugs in animals and humans: biology and behavior. *Journal of Addictive Diseases*.

[66] Lyoo I. K., Pollack M. H., Silveri M. M. (2006). Prefrontal and temporal gray matter density decreases in opiate dependence. *Psychopharmacology*.

[67] Schlaepfer T. E., Lancaster E., Heidbreder R. (2006). Decreased frontal white-matter volume in chronic substance abuse. *International Journal of Neuropsychopharmacology*.

[68] Delisi L. E., Bertisch H. C., Szulc K. U. (2006). A preliminary DTI study showing no brain structural change associated with adolescent cannabis use. *Harm Reduction Journal*.

[69] Massat I., Kocabas N. A., Crisafulli C. (2011). COMT and age at onset in mood disorders: a replication and extension study. *Neuroscience Letters*.

[70] Fiori L. M., Wanner B., Jomphe V. (2010). Association of polyaminergic loci with anxiety, mood disorders, and attempted suicide. *PLoS ONE*.

[71] Le-Niculescu H., Levey D. F., Ayalew M. (2013). Discovery and validation of blood biomarkers for suicidality. *Molecular Psychiatry*.

[72] Lichtenstein P., Yip B. H., Björk C. (2009). Common genetic determinants of schizophrenia and bipolar disorder in Swedish families: a population-based study. *The Lancet*.

[73] Moskvina V., Craddock N., Holmans P. (2009). Gene-wide analyses of genome-wide association data sets: Evidence for multiple common risk alleles for schizophrenia and bipolar disorder and for overlap in genetic risk. *Molecular Psychiatry*.

[74] Purcell S. M., Wray N. R., International Schizophrenia Consortium (2009). Common polygenic variation contributes to risk of schizophrenia and bipolar disorder. *Nature*.

[75] van Snellenberg J. X., de Candia T. (2009). Meta-analytic evidence for familial coaggregation of schizophrenia and bipolar disorder. *Archives of General Psychiatry*.

